# Summary of Guidelines for Identifying and Risk-Stratifying Patients with Metabolic Dysfunction-Associated Steatotic Liver Disease: A Primer for Family Physicians

**DOI:** 10.3390/diagnostics16121854

**Published:** 2026-06-15

**Authors:** Mitchell P. Wilson, Abdel-Aziz Shaheen, Victoria Leung, An Tang, Andreu F. Costa, Casey Hurrell, Gavin Low

**Affiliations:** 1 Department of Radiology and Diagnostic Imaging, University of Alberta, 2B2.41 WMC, 8440-112 Street NW, Edmonton, AB T6G 2B7, Canada; 2 Division of Gastroenterology, Department of Medicine, University of Calgary, 3280 Hospital Drive NW, Calgary, AB T2N 4Z6, Canada; 3 Department of Family Medicine, Faculty of Medicine and Dentistry, University of Alberta, 6-10 University Terrace, Edmonton, AB T6G 2T4, Canada; 4 Department of Radiology, Radiation Oncology and Nuclear Medicine, Université de Montréal, 1000 R. Saint-Denis, Montreal, QC H2X 0C1, Canada; 5 Department of Diagnostic Radiology, Faculty of Medicine, Dalhousie University, Halifax, NS B3H 4R2, Canada; 6 Canadian Association of Radiologists, 220 Ave. Laurier #1120, Ottawa, ON K1P 6E6, Canada

**Keywords:** liver, MASLD, NAFLD, risk stratification, FibroScan, transient elastography, shear wave elastography, MR elastography

## Abstract

Multiple North American and European societies now endorse a combined serological and imaging-based clinical care pathway for non-invasive risk stratification of patients with metabolic dysfunction-associated steatotic liver disease (MASLD). A multidisciplinary group of Canadian radiologists, hepatologists, family physicians, and other health professionals have recently published consensus guidelines for identification and risk stratification of patients with suspected MASLD. Screening should be performed with the FIB-4 score, and those with an indeterminate FIB-4 (between 1.32.67) should undergo imaging-based liver stiffness evaluation either with transient elastography (FibroScan), ultrasound shear wave elastography, or magnetic resonance elastography as a second step. While the implementation of these techniques for measuring liver stiffness differ, there is no clinically significant difference in their diagnostic performance. This narrative review, intended for Family Physicians, summarizes recommendations for serological investigations and imaging modalities of liver steatosis and stiffness. Practical guidance includes an algorithm with thresholds. We discuss current challenges and future directions of risk-stratifying patients with MASLD in the community.

## 1. Introduction

Metabolic dysfunction-associated steatotic liver disease (MASLD), previously known as non-alcoholic fatty liver disease (NAFLD), is the most common cause of chronic liver disease worldwide with a rising prevalence and mortality rate [1,2,3,4,5]. MASLD is a type of steatotic liver disease, which is mainly related to metabolic disorders such as diabetes mellitus and obesity. MASLD is diagnosed by the presence of hepatic steatosis and the presence of any one of the following five cardiometabolic risk factors: elevated BMI or waist circumference, type 2 diabetes, hypertension, hypertriglyceridemia, and dyslipidemia [6]. This paper focuses on MASLD excluding alcohol-related conditions like metabolic-and-alcohol-related liver disease (Met-ALD) and alcohol-related liver disease (ALD).

MASLD itself is a spectrum of disease, ranging from isolated abnormal liver fat deposition to the presence of inflammation (a hallmark feature of abnormal hepatocellular ballooning and lobular inflammation known as metabolic dysfunction-associated steatohepatitis [MASH] (formerly termed NASH)), which can then progress to hepatic fibrosis, which is staged from F1 to F4 (where F4 is also termed cirrhosis) [2]. Importantly, it is the presence of hepatic fibrosis, not the degree of fat deposition or inflammation that is associated with worse outcomes. Patients with significant (defined as ≥F2), advanced hepatic fibrosis (≥F3) and cirrhosis (F4) have progressively worse survival [7,8,9]. In North America, it is estimated that 30% of the general population have MASLD, 6% have MASH, and 3% have both MASH and significant (≥F2) fibrosis, which carries an increased risk of liver-related events such as decompensated cirrhosis, liver failure, and hepatocellular carcinoma [6,10,11,12].

The purpose of risk-stratifying patients with MASLD is to identify patients most at risk for liver-related events, and implement more aggressive lifestyle and medical interventions to prevent and potentially reverse early stages of fibrosis [1,13,14,15]. Although liver biopsy has served as the traditional reference standard for staging patients with liver disease, it carries several limitations. It is an impractical tool to risk-stratify the large subgroup of the population with MASLD. Biopsy is only available at particular centers, carries high costs and is associated with risks of complications such as hemorrhage and non-representative samples or sampling errors, which may lead to discrepant results in staging of liver fibrosis. However, biopsy is a valuable tool reserved for select individuals, such as those with indeterminate liver stiffness measurements, liver disease of unknown etiology, or discordant serological and imaging results.

There has been substantial research in developing non-invasive approaches to risk-stratify patients with MASLD and studies have led to North American and European guidelines to identify, risk-stratify, and manage patients with MASLD [1,16,17,18,19]. These guidelines all implement a combination of serological and image-based investigations to guide management. Recently, a multidisciplinary group of Canadian radiologists, hepatologists, family physicians, and other health professionals adapted and endorsed principles of these guidelines to the Canadian setting [16,20,21]. The purpose of this review is to provide an overview of serological and imaging investigations to primary care practitioners for risk-stratifying patients with suspected MASLD, to review Canadian guideline recommendations, and discuss current challenges and future directions of risk-stratifying patients with MASLD.

## 2. Serological Tests

In isolation, direct and indirect blood serum biomarkers of liver disease lack sufficient accuracy to risk stratify patients with MASLD [17,22]. Several combination scores incorporating clinical variables and indirect or direct serological biomarkers of liver disease have been evaluated with several having shown improved accuracy for identifying patients with advanced hepatic fibrosis (≥F3) [17]. Combined clinical and indirect biomarkers are most common as they are non-proprietary and cost effective. The most common blood-based risk stratification tools and their pooled performance are shown in Table 1. These tools have adequate sensitivities and negative predictive values for ruling out disease but lack sufficient positive predictive value in low prevalence disease cohorts [17]. Because of its low cost, widespread availability and robust evidence to date, the FIB-4 score has become the most widely recognized serum biomarker for risk-stratifying patients with MASLD [17,23]. FIB-4 has been incorporated into multiple clinical guidelines with thresholds of <1.3 to identify low risk patients and >2.67 to stratify high risk patients for advanced hepatic disease [1,16,17,19]. A high risk FIB-4 threshold of >2.67 to “rule in” disease has been shown to have a specificity of 96% for detecting advanced hepatic fibrosis on pooled analysis in this patient population [24].

There are some important limitations of the FIB-4 score. Whereas FIB-4 is accurate in patients aged between 36–65, its accuracy decreases at extremes of age. Some authors consider that FIB-4 is not suitable to risk stratify patients ≤35 years of age and that a low-risk threshold of 2.0 rather than 1.3 should be used for patients ≥65 years [25]. The appropriate use of FIB-4 in adult patients ≤35 years of age and specified thresholds for patients aged ≥65 should be determined based on approved regional practices by liver specialists. Further, alteration of indirect biomarkers underpinning the FIB-4 calculation for reasons other than liver fibrosis can render the results invalid. Examples include patients with thrombocytopenia, active alcohol use or acute liver inflammation which can all falsely elevate the FIB-4 score, and chronic renal disease which can falsely lower the FIB-4 score [17].

## 3. Imaging Tests

### 3.1. Detecting Hepatic Steatosis and Liver-Related Complications

Steatosis can be diagnosed on ultrasound (US), non-contrast CT, or MRI [20]. Family physicians and radiologists must recognize that, while imaging often detects steatosis, it cannot determine its cause and therefore cannot diagnose patients with MASLD. This requires a detailed history, physical exam, and serological testing, which includes a review of medications and other illnesses [26].

Conventional B-mode US has a sensitivity and specificity of 85% (95% CI, 80–89%) and 94% (95% CI, 87–97%), respectively, for detecting moderate to severe steatosis but a sensitivity of only 70% (95% CI, 63–77%) for detecting mild steatosis [27,28]. Quantitative US techniques utilize alterations in the changing physical properties of ultrasound waves to detect and grade hepatic steatosis. Although these quantitative techniques have shown promise, they are not widely used in current practice [29,30]. Non-contrast CT has a sensitivity of 82% (95% CI, 67–91%) and specificity of 94% (95% CI, 88–97%) for detecting moderate-to-severe hepatic steatosis [31]. Contrast-enhanced CT lacks sufficient data to reliably detect hepatic steatosis, but may be specific for detecting moderate to severe steatosis in specific instances [20]. Finally, MRI is an excellent tool for detecting and grading hepatic steatosis, whether with routinely available sequences or with specialized sequences for accurate fat quantification [20]. Although quantification of hepatic steatosis is of research interest, the severity of hepatic steatosis does not predict the risk of steatohepatitis, fibrosis, or survival [7,9]. Hence, quantification of hepatic steatosis is currently not incorporated in major guidelines.

Conventional US, CT, and MRI can also assess for morphological signs of advanced fibrosis, such as a nodular texture, surface lobulations, features of portal hypertension (such as splenomegaly, ascites, and portosystemic collaterals), and the presence of hepatocellular carcinoma. These morphological features of advanced fibrosis and portal hypertension are helpful when present, but the absence of these imaging features does not exclude advanced fibrosis or portal hypertension—hence the need for liver stiffness measurements as outlined below. In patients with any type of steatotic liver disease, there is no indication for imaging to detect or grade hepatic steatosis, or to identify features of advanced fibrosis or portal hypertension [20]. There is a lack of proven benefit [20], and such practice would place unmanageable demands on imaging resources, waitlists, and healthcare costs.

### 3.2. Liver Stiffness Measurements

In patients with indeterminate FIB-4 score, further assessment with liver stiffness measurements (LSMs) is indicated. LSMs can be acquired using multiple different techniques including vibration-controlled transient elastography (TE, commercialized as FibroScan), shear wave elastography (SWE), and magnetic resonance elastography (MRE). SWE can be further subdivided into point SWE (pSWE) and 2-dimensional SWE (2D-SWE). Each modality uses different physical properties and vendor-specific propriety software to acquire an LSM. LSMs have been shown to correlate with the stage of hepatic fibrosis [32]. While TE is the most well studied, no substantial differences in performance have been identified between modalities for detecting significant (≥F2) and advanced (≥F3) hepatic fibrosis [24]. As such, each modality is accepted by multiple guidelines for risk-stratifying patients with MASLD [1,16,18,19,32]. However, there is some evidence to suggest that thresholds for pSWE may be lower and less sensitive than 2D-SWE, and 2D-SWE is recommended for ultrasound-based reporting when both are available [16,24,33]. Details regarding the physical properties and performance values for each LSM tool are shown in Table 2.

There are individual benefits of each LSM assessment modality. First, TE is the most well-validated tool and is often incorporated into existing liver specialist services; this allows for simultaneous diagnosis and management in a specialized setting. The benefits of SWE include real-time anatomic assessment of the liver parenchyma, which allows the sonographer or radiologist to avoid structures such as vessels and focal liver lesions, and obtain optimal sampling of the liver tissue using a standardized technique. Anatomical assessment with SWE has the added benefit of simultaneous evaluation for visual signs and complications of advanced fibrosis. SWE will likely become more available than TE as more diagnostic imaging providers integrate SWE technology into existing and new ultrasound machines. Moreover, as an imaging-based modality available in select diagnostic radiology departments, it has been shown that SWE is a cost-effective method to evaluate patients with MASLD and indeterminate FIB-4 scores, while simultaneously reducing the number of hepatology referrals [23].

MRE provides the most robust assessment of the entire liver, which reduces the risk of under sampling due to liver disease heterogeneity. Co-existing liver disease can also be assessed with MRE, including simultaneous quantification of hepatic steatosis and liver iron deposition, the so-called liver “triple screen” [34,35]. However, MRE is less widely available, requires more radiologist training for interpretation, and is more costly than TE and SWE. As such, MRE is best used on a case-by-case basis, such as when TE and SWE are non-diagnostic or in the case of discordance between them, which can occur with obesity, or when other liver diseases are suspected, such as increased iron deposition or biliary disease [36].

### 3.3. Guideline Recommendations

The 2021 American Gastroenterological Association (AGA) guidelines [19] recommended risk stratification in patients with type 2 diabetes, two or more metabolic risk factors, or incidentally identified hepatic steatosis or elevated aminotransferases. These patients are evaluated with clinical history and serological investigations including a FIB-4 score, which stratified patients into low risk (FIB-4 < 1.3), intermediate risk (FIB-4 between 1.3 and 2.67), and high risk (FIB-4 > 2.67) for developing liver-related complications. Patients at low risk undergo routine surveillance in 2–3 years unless clinical change. Intermediate risk patients were further stratified with a LSM (TE or SWE) using a threshold <8 kPa to define low risk patients; indeterminate risk patients were defined as 8–12 kPa and high risk patients were defined as >12 kPa. Both patients with indeterminate risk or those at high risk warrant a liver specialist consultation while indeterminate risk patients may also benefit from a MRE or liver biopsy.

In 2024, the American Association for the Study of Liver Diseases (AASLD) and the European Association for the Study of the Liver (EASL) separately offered similar guideline recommendations [1,17,18]. The FIB-4 thresholds for patients with MASLD are the same, with a <8 kPa threshold using TE or SWE utilized to define low risk patients in the indeterminate category (FIB-4 between 1.32.67). Low risk patients should undergo follow up, for which EASL recommends follow up every 1–3 years. For patients with LSMs above the 8 kPa threshold, liver specialist consultation is advised to guide further management. All three guidelines support the use of TE or SWE to define the LSM. MRE is reserved as a supplementary investigative tool for indeterminate risk patients or when the LSM is non-diagnostic using TE or SWE. Additionally, EASL guidelines have also supported Enhanced Liver Fibrosis (ELF), a direct serological biomarker, as an alternative second step examination to TE or SWE [1]. Although ELF is available in many jurisdictions, it is not often publicly funded and thus not frequently used in many locations around North America and Europe.

**Figure 1:**
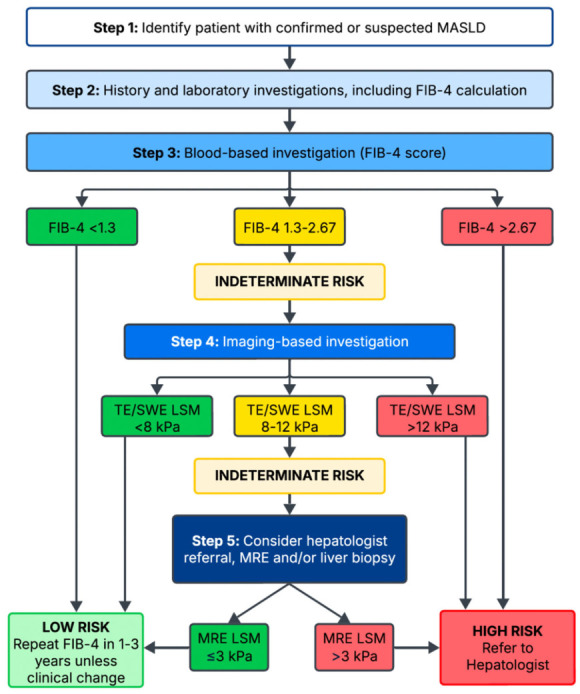
Recent multi-specialty Canadian Association of Radiologists MASLD Working Group strategy for population screening and risk stratification of MASLD patients. The Working Group is a multidisciplinary group consisting of radiologists, hepatologists, and family physicians. Reproduced with permission from Wilson et al. [16]. Patients who may warrant screening and risk stratification for MASLD include patients with type 2 diabetes mellitus, 2 or more metabolic risk factors (including central obesity, high triglycerides, low HDL cholesterol, hypertension, prediabetes, or insulin resistance), elevated aminotransferases, and/or incidental imaging features suggestive of hepatic steatosis. FIB-4 = [Age (years) × AST (U/L)]/[Platelet count (109/L) × √ALT (U/L)]. MASLD = Metabolic dysfunction-associated steatotic liver disease; TE = Vibration-controlled transient elastography; SWE = Shear wave elastography (may either be point or 2D shear wave elastography); LSM = Liver stiffness measurement; MRE = Magnetic resonance elastography.

In 2025, a Canadian multidisciplinary group of radiologists, hepatologists, family physicians, and other health professionals reviewed, synthesized, and endorsed a similar multi-level non-invasive approach [16]. The strategy is outlined in Figure 1. The risk stratification strategy is based on five steps. The first step is to identify patients with confirmed or suspected MASLD based on the presence of steatosis and a cardiometabolic risk factor. The second step requires history and laboratory investigations to exclude alternative causes of steatotic liver disease. The third step stratifies the risk based on FIB-4 scores. The fourth step evaluates liver stiffness measurements using ultrasound-based elastographic techniques such as TE and SWE. The fifth step applies to patients with indeterminate risk who may require referral to a hepatologist, MRE, and/or liver biopsy to resolve discrepancies.

## 4. Challenges and Future Directions

An individual participant data meta-analysis of over 5000 patients with MASLD with a histological reference standard found that a combination of FIB-4 threshold < 1.3 and LSM < 8 kPa results in a sensitivity of 65% (95% CI, 62–68%) and negative predictive value of 86% (95% CI, 85–87%) for excluding advanced hepatic fibrosis [37]. As the multi-step thresholds now endorsed by multiple societies gain further adoption in routine clinical practice, studies evaluating the diagnostic performance, cost-effectiveness, and patient outcomes are necessary. This will likely require large multi-institutional trials, ideally with prospective study designs and a composite reference standard based on clinical, serological, imaging, and histological findings to recognize a lower prevalence of significant and advanced fibrosis in the community setting.

It is possible that future research will refine patient pathways and threshold scores. For example, the currently recommended lower FIB-4 threshold of 1.3 was originally proposed in a study of 541 patients with MASLD, which showed a sensitivity of 74% and a negative predictive value of 90% for excluding advanced hepatic fibrosis [38]. While this has since been validated across multiple studies, the accuracy of this result needs to be evaluated in the community setting. Recent meta-analyses have suggested that a lower FIB-4 threshold is needed to achieve a 90% sensitivity for excluding advanced hepatic fibrosis [24,37]. The ideal real-world threshold can be evaluated in consort with prospective studies noted above and will be reassessed with future guideline revisions.

It is expected that, as adoption of risk stratification of MASLD expands in the community setting, SWE will become more widely available. More studies are needed to evaluate the diagnostic performance of SWE. This includes not only the performance of SWE itself, but also head-to-head studies comparing TE, pSWE, and 2D-SWE, as well as comparison between vendors, and potentially other patient subgroups, as predictive values are impacted by disease prevalence. While the utility of a modality and vendor-agnostic LSM threshold for SWE is favored for practical reasons, future guideline revisions with modality and/or vendor specific thresholds may be necessary [16,24].

Finally, the utility of these guidelines for surveillance will need to be refined over multiple years. Guidelines recommend a range of surveillance between 13 years with individual surveillance periods based on regional practice and liver specialist recommendations. The frequency of surveillance will need to be monitored, with possible shorter surveillance periods for higher risk groups, such as those with type 2 diabetes [1]. Further, the utility of SWE thresholds for surveillance will need to be further validated. Currently, best practice recommendations include a clinically significant change of 10%, ideally performed on the same device as was used previously [16,39]. Studies have shown that among 50% of screened cases, TE has approximately 20% variation (≥2 fibrosis stages) [40]. Similar findings have also been observed within SWE systems [41]. There is likely variability between operators, vendors and individual machines, and this variability needs to be evaluated.

## 5. Conclusions

MASLD is the most common cause of chronic liver disease, and its prevalence and mortality rates are increasing. MASLD is a massive health problem that requires evidence-based care and coordination of efforts by multiple specialties. Multiple societal guidelines, including a recent multidisciplinary guidance across North America and Europe, now recommend a combined serological followed by imaging-based pathway for risk-stratifying patients with MASLD. Patients with MASLD should be initially screened with a FIB-4 score, and depending on results, either undergo routine surveillance, further evaluation with LSM, or liver specialist referral. LSMs can be performed with TE, SWE or MRE. While no clinically significant differences in performance have been identified between these modalities, the technology underpinning each is fundamentally different, and this represents an area of active investigation. Based on cost and accessibility, SWE is recommended over TE and MRE, and, where available, 2D-SWE is recommended over pSWE. Because of its higher cost and lower availability, MRE should be reserved as a troubleshooting tool, for example in indeterminate risk patients, non-diagnostic TE or SWE, or when other liver diseases are suspected. This field is rapidly evolving, and it is expected that guideline revisions will be frequently required in response to developments in the literature.

## Figures and Tables

**Table 1:** Components of calculation and pooled lower threshold sensitivity and specificity results for detecting advanced hepatic fibrosis in patients with MASLD using common blood-based combination investigations.

Test	Components of Calculation *	Lower Thresholds **	≥F3 Sensitivity **	≥F3 Specificity **
APRI	AST, platelet count	0.5	78%	65%
FIB-4	Age, AST, ALT, platelet count	1.3	82%	64%
NFS	Age, BMI, impaired fasting glucose, AST, ALT, platelet count	−1.455	75%	NR

* Components of calculation include both clinical variables and indirect markers of liver disease. No direct markers are included in these calculations. ** Lower “rule out” thresholds and performance values are adapted from meta-analysis results by Sterling et al. [17]. Lower threshold results have been provided as these tests have been deemed to have adequate sensitivity and negative predictive values in existing guidelines [1,16,17,19]. FIB-4 = Fibrosis-4 Index; APRI = AST to platelet ratio index; NFS = NAFLD fibrosis score; AST = aspartate aminotransferase; ALT = alanine aminotransferase; BMI = body mass index.

**Table 2:** Vendors, pooled performance values and physical properties of different liver stiffness measurement tools.

		≥F2			≥F3			
LSM Technique	Vendors	Thresholds **	Sensitivity (95% CI) **	Specificity (95% CI) **	Thresholds **	Sensitivity (95% CI) **	Specificity (95% CI) **	Physical Properties
TE	FibroScan (Echosens)	7 (6.5–7.4) kPa	76% (70–82%)	73% (68–78%)	10 (9.5–10.4) kPa	82% (76–87%)	79% (70–85%)	External mechanical vibration of the soft tissues which generates a quantitative evaluation of liver stiffness without structural details of the liver. Measurements are repeated 10 times across the expected region of the liver, typically at the mid-axillary line. Measurements must have an interquartile range/median ≤30% to report.
pSWE	Phillips, Samsung, Siemens *	1.2 (1–1.3) m/s	85–90%	36–90%	1.5 (1.4–1.53) m/s	70% (58–80%)	92% (89–94%)	Ultrasound-based proprietary vendor-specific software which uses a focused short-duration acoustic radiation force impulse (ARFI) to generate a shear wave as a single measurement within the liver. Measurements are repeated 10 times across the liver. Measurements must have an interquartile range/median ≤30% to report.
2D-SWE	Canon, GE, Siemens, SuperSonic Imagine, Toshiba, Ultrasign *	7–7.7 kPa	85% (71–92%)	79% (61–90%)	8–8.9 kPa	90% (85–93%)	79% (62–89%)	Ultrasound-based proprietary vendor-specific software which uses a focused short-duration acoustic radiation force impulse (ARFI) to generate a shear wave within a 2-dimensional box in the liver. Measurements taken from within the image box and are repeated 5–10 times across the liver. Measurements must have an interquartile range/median ≤30% to report.
MRE	GE, Philips, Siemens *	3.2–3.6	78% (65–87%)	90% (84–94%)	3.7 (3.6–3.9) kPa	82–93%	90–95%	External mechanical vibration of the soft tissues which generates a quantitative evaluation of liver stiffness across an entire cross section of the liver. Measurements are repeated at four different levels of the liver and results are averaged for reporting. This serves as the most accurate measure of the entire liver.

* Non-exhaustive alphabetical list of known pSWE, 2D-SWE, and MRE vendors. ** Thresholds and performance values are adapted from meta-analysis results by Duarte-Rojo et al. [32]. Presented performance values are for detecting significant (≥F2) and advanced (≥F3) hepatic steatosis in patients with known MASLD. LSM = liver stiffness measurement; TE = transient elastography; pSWE = point shear wave elastography; 2D-SWE = 2-dimensional shear wave elastography; MRE = magnetic resonance elastography; GE = General Electric.

## Data Availability

No new data were created or analyzed in this study. Data sharing is not applicable to this article.
